# The Gα GOA-1/GNAO1 non-autonomously promotes germline stem cell quiescence in feminized *C. elegans* hermaphrodites from the gonadal sheath cells

**DOI:** 10.17912/micropub.biology.002250

**Published:** 2026-08-17

**Authors:** Armi M Chaudhari, Minh Thu Nguyen, Patrick Narbonne

**Affiliations:** 1 Département de Biologie Médicale, University of Quebec at Trois-Rivières, Trois-Rivières, QC, Canada

## Abstract

Stem cell proliferation rates must be precisely regulated as insufficient proliferation leads to tissue loss, whereas excessive proliferation causes tumorigenesis and cancer. A homeostatic balance is therefore achieved through feedback loops that adjust stem/progenitor cell proliferation rates to match the demand for their differentiated progeny. While such a homeostatic feedback mechanism adjusts germline stem cell (GSC) proliferation rates to oocyte needs in the *Caenorhabditis elegans* adult hermaphrodite germline, its inner workings are incompletely understood. Here we show that the Gα GOA-1 /GNAO1 is required specifically in the gonadal sheath cells to non-autonomously promote GSC quiescence in spermless hermaphrodites. Given that dysregulation of G protein signalling is frequently observed in human cancers, Gα dependent homeostatic control of stem cell proliferation may represent a conserved tumour suppressive mechanism.

**Figure 1. GOA-1 suppresses GSC proliferation from the gonadal sheath cells:** **(A)** Model for homeostatic regulation of germline stem/progenitor cell (GSC) proliferation rates in adult *C. elegans* hermaphrodites. GSCs located in the distal progenitor zone (PZ) generate daughters that, upon exiting the niche, differentiate into oocytes. Major sperm proteins (MSPs) released by sperm stimulate oocyte maturation and ovulation via G-protein coupled receptors and cAMP signalling in the gonadal sheath cells (Govindan et al., 2006, 2009) to establish oocyte demand. A strong oocyte demand in turn promotes MPK-1 /ERK-dependent GSC proliferation (Lee et al., 2007; Narbonne et al., 2017; Robinson-Thiewes et al., 2021). **(B-E)** The number of **(B, D)** oocytes in diakinesis per gonad arm and **(C, E)** the GSC mitotic index of day-1 adult (A1) hermaphrodites of the indicated genotypes were scored. Each bar represents the mean ± standard deviation, and each point represents the value from a single gonad arm; one or both arms per individual were scored. Triple asterisks indicate statistical significance *vs* the wild-type (P<0.001). Statistical analyses used a one-way ANOVA followed by Tukey's multiple comparisons for **(B, D)** and the Kruskal–Wallis test followed by Dunn's multiple comparisons for **(C, E)** . ns, not significant. **(F)** Representative differential interference contrast (DIC) micrographs of proximal gonads of A1 hermaphrodites of the indicated genotypes. The bottom panel shows the epifluorescence signal from the sheath-expressed *P lim-7 :: GOA-1 [N-term]::GFP:: GOA-1 [C-term] (* abbreviated as *P lim-7 :: GOA-1 ::GFP)* construct as a green overlay. **(G)** The sheath and spermathecal (Sp) expression patterns and levels from the single-copy *P goa-1 :: GOA-1 ::GFP* insertion (Kumar et al., 2021) were similar in A1 (top) control and (bottom) feminized *fog-1 (lf)* mutants. **(H)** The (top) *oma-1 (ø); oma-2 (lf) (n=18)* double mutants usually maintain a state of relatively stable oocyte hyperplasia beyond A2 while all the (bottom) *goa-1 (lf); oma-1 (ø); oma-2 (lf) (n=22)* triples have hyperaccumulated oocytes by then. A tumour penetrance percentage is shown as a coloured vertical bar on the right (blue, stable hyperplasia; red, oocyte hyperaccumulation/differentiated tumour). **(I)** Example of a younger tumorous *goa-1 (lf); oma-1 (ø); oma-2 (lf)* mutant, slightly after it progressed beyond hyperplasia and clearly started to hyperaccumulate large oocyte-like differentiated germ cells. ( **F-I** ) Yellow dotted lines mark the region occupied by oocyte-like cells. Scale bars, 50 μm. **(H-I)** All strains contain *cpSi42 [Pmex-5::mNeonGreen::PLCδ-PH:: tbb-2 3'UTR + unc-119 (+)]* , marking germ membranes with green fluorescence. ( **B-H** ) Alleles, *gsa-1 ( ce94 )gf* , *fog-1 ( q253 )ts, fog-1 ( q785 )ø* , *goa-1 ( n1134 )lf* , *vsSi32 [ goa-1 ::GFP + unc-119 (+)], narEx166 [pCAM37(P lim-7 :: GOA-1 [N-term]::GFP:: GOA-1 [C-Term]); Pmyo-3::mCherry], oma-1 ( zu405te33 )ø, oma-2 ( te51 )lf.
*

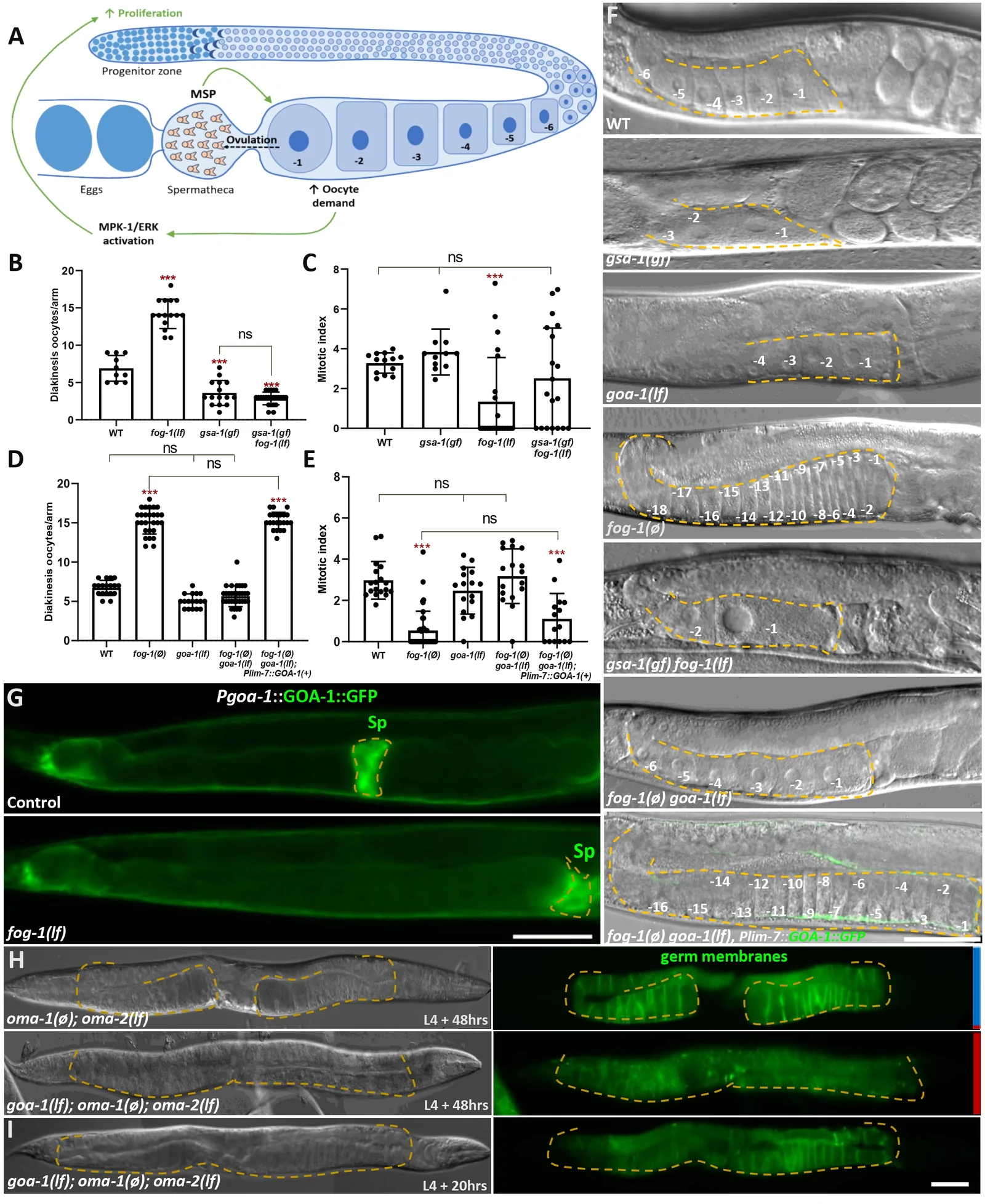



## Description

Stem cells must divide often enough to replace cells lost to turnover and be able to increase their output in response to injuries. Yet their proliferation must also be constrained to prevent tissue overgrowth and tumorigenesis when differentiated cell turnover is reduced. This balance is achieved through homeostatic feedback loops that monitor differentiated tissue status and modulate stem/progenitor cell proliferation accordingly. At the molecular level, G protein-coupled receptor (GPCR) signalling has emerged as a key regulator of stem cell proliferation and homeostasis across diverse systems. GPCRs mediate stem cell responses to extracellular cues through heterotrimeric G proteins, with stimulatory (Gαs) and inhibitory (Gαi/o) pathways modulating stem cell division rates, self-renewal, and differentiation in multiple contexts (Kobayashi et al., 2010; Layden et al., 2010; Pedro et al., 2020). In *Drosophila* males for instance, germline stem cells (GSCs) increase their division frequency in response to mating-induced sperm depletion through a germ-autonomous G protein-dependent mechanism, directly demonstrating that GPCR signaling can couple stem cell output to physiological demand (Malpe et al., 2020). However, whether G protein signalling can non-autonomously contribute to tissue homeostasis has remained unclear.

The *Caenorhabditis elegans* germline provides a powerful *in vivo* system to elucidate how stem cell proliferation homeostasis is controlled. Hermaphrodites produce a finite number of sperm during larval development before shifting irreversibly to oocyte production in adults, with oogenesis continuing at a fast pace until sperm reserves are depleted ( Fig. 1A ). As sperm-secreted major sperm proteins (MSPs) actively stimulate meiotic maturation and ovulation (Miller et al., 2001), once sperm reserves are depleted, arrested oocytes accumulate until a homeostatic feedback signal suppresses GSC proliferation/differentiation to stabilize a state of oocyte hyperplasia (Morgan et al., 2010; Narbonne et al., 2015, 2017; Cinquin et al., 2016; Valet & Narbonne, 2022).

The antagonistic inhibitory Gαo/i GOA-1 /GNAO1 and stimulatory Gαs GSA-1 /GNAS1 have been identified as critical regulators of MSP/ovulation signalling, where the loss of *goa-1* results in spontaneous ovulation in the absence of sperm/MSPs while conversely, the loss of *gsa-1* activity prevents ovulation, even in the presence of sperm/MSPs (Govindan et al., 2006, 2009). Interestingly, a *goa-1* loss-of-function (lf) further impaired GSC proliferation homeostasis in feminized *fog-1* (ø) spermless hermaphrodites (Narbonne et al., 2015). However, and especially since the loss of *goa-1* is pleiotropic, also affecting behaviour and locomotion (Ségalat et al., 1995; Mendel et al., 1995), it remained unclear if the disruption of GSC proliferation homeostasis specifically resulted from the very same role that *goa-1* has in MSP signal transduction.

Germ-specific RNAi and elegant mosaic analyses unequivocally supported a requirement for *goa-1* and *gsa-1* in the somatic gonad for the regulation of oocyte maturation and ovulation (Govindan et al., 2006, 2009; Korswagen et al., 1997). Further, a single mosaic individual for *acy-4* , encoding the adenylate cyclase acting downstream of these Gα proteins, more specifically indicated a sheath cell requirement for this gene in ovulation control (Govindan et al., 2009). However, whether *gsa-1* and *goa-1* activities are also acting specifically within the gonadal sheath cells, rather than in another somatic gonad tissue, has remained unconfirmed.

While the gonadal sheath and spermathecal cells derive from a common somatic precursor, GSA-1 was recently implicated in the control of spermathecal contractility through a molecular cascade that overlaps with MSP signalling (Castaneda et al., 2020). Given this shared developmental origin and our recent discovery that spermathecal neck contractility was key in preventing ovulation in feminized hermaphrodites (Deng et al., 2025), we deemed it important to determine whether sheath-specific *goa-1* activity was sufficient to regulate both ovulation and germline stem cell proliferation homeostasis in feminized hermaphrodites.

We first reasoned that if GOA-1 and GSA-1 have opposing activities within both MSP signalling and homeostatic GSC proliferation control, activating *gsa-1* with a gain-of-function (gf) allele should prevent GSC downregulation in feminized hermaphrodites, like the loss of *goa-1* . Consistent with this, *gsa-1* (gf) prevented oocyte accumulation in feminized hermaphrodites and blocked the downregulation of GSC proliferation ( Fig. 1B- C). To directly test the gonadal sheath requirement for *goa-1* , we used a standard extrachromosomal array transgene (Mello et al., 1991) to restore *goa-1* specifically in this tissue within feminized *goa-1* (lf) mutants, using the sheath-specific *lim-7* promoter (Voutev et al., 2009). As conventional N- or C-terminal tagging of Gα proteins can impair their function (Hynes et al., 2004), Kumar et al. inserted a GFP tag between residues 117-118 of GOA-1 to create a functional single-copy P *goa-1* :: GOA-1 ::GFP insertion (Kumar et al., 2021). We used this strain as a starting point to generate a P *lim-7* :: GOA-1 [N-term]::GFP:: GOA-1 [C-term] extrachromosomal array, abbreviated as P *lim-7* :: GOA-1 ::GFP. The resulting transgene effectively rescued both oocyte accumulation and GSC MI downregulation in *fog-1* (ø) *goa-1* (lf) hermaphrodites ( Fig. 1D- F). We also examined whether sheath or spermathecal expression from a P *goa-1* :: GOA-1 ::GFP single-copy transgene (Kumar et al., 2021) were altered by feminization, but no obvious changes were observed ( Fig. 1G ). Together with earlier findings (Govindan et al., 2006, 2009), our results provide strong evidence that GOA-1 acts specifically within the gonadal sheath to prevent spontaneous ovulation events in the absence of sperm/MSP to permit oocyte accumulation, and at the same time yet indirectly, to non-autonomously promote homeostatic downregulation of GSC proliferation.

Similar to the loss of GOA-1 , loss of the AMPK α-catalytic subunit AAK-1 caused spontaneous ovulation and prevented homeostatic GSC downregulation in feminized hermaphrodites (Narbonne et al., 2017). The specific suppression of oocyte maturation through the inactivation of OMA-1 ,2 proteins (Detwiler et al., 2001) restored oocyte accumulation in *aak-1* (ø) mutants but failed to reinstate homeostatic GSC downregulation, resulting in the formation of benign differentiated germline tumours akin to hamartomas, in which differentiated oocyte-like cells hyperaccumulate in a disorganized manner as a consequence of homeostatically unchecked GSC proliferation (Ali & Mulita, 2023; Narbonne et al., 2017; Valet & Narbonne, 2022). We find that likewise, reduced *goa-1* activity in an *oma-1* (ø); *oma-2* (lf) background provokes a hyperaccumulation of oocyte-like cells ( Fig. 1H- I). The similarity of the *goa-1* (lf) and *aak-1* (ø) phenotypes raises the intriguing possibility that *aak-1* may essentially permit sheath Gα signals to suppress oocyte maturation and ovulation in the absence of sperm.

Altogether, our results reveal that the stimulatory Gα GOA-1 /GNAO1 ensures homeostatic GSC downregulation in spermless adult hermaphrodite *C. elegans* non-autonomously from the gonadal sheath cells, likely through its known roles in MSP responses and ovulation control. G-protein signalling may therefore non-autonomously regulate stem cell proliferation rates in various organisms and tissues in addition to having cell autonomous roles.

## Methods

***C. elegans* strains and maintenance**

*C. elegans* strains were maintained at 15 ^o^ C on standard nematode growth medium (NGM) seeded with *E. coli* ( OP50 ) unless otherwise indicated. The Bristol ( N2 ) isolate served as wild-type throughout (Brenner, 1974). Table 1 provides a complete list of the *C. elegans* strains and reagents used in this work.

**Plasmid and transgenic strain**

We used the Gibson method (Gibson et al., 2009) for plasmid assembly; primers are listed in Table 1. To achieve sheath-specific *goa-1* rescue, the LX2060 strain carrying a single-copy insertion of a GOA-1 ::GFP translational fusion driven by 5 kb of *goa-1* promoter (Kumar et al., 2021) was used to amplify the *goa-1* N-term::GFP:: *goa-1* C-term genomic sequence (~4.5 kb). The resulting fragment was inserted downstream of the *lim-7* promoter in a vector derived from pGC240 (Voutev et al., 2008). The integrity of the resulting pCAM37 plasmid was confirmed by XbaI restriction digestion. The microinjection mix consisted of three plasmids: pCAM37 at 50 ng/µL, pCFJ104 at 5 ng/µL (Frøkjær-Jensen et al., 2008) and pKSII as carrier DNA at 150 ng/µL. The mixture was microinjected into the germline syncytium of wild-type hermaphrodites (Mello et al., 1991) to establish and select an array that was subsequently crossed into the *fog-1* (ø) *goa-1 (lf)* double mutant strain for assaying its rescuing activity.

**Imaging and image processing**

All hermaphrodites used for imaging or scoring were synchronized by picking late-L4 stage larvae, based on vulva development (Seydoux et al., 1993), to a new plate that was upshifted to 25 °C for an additional 24 hours to generate day-1 adults (referred to as A1) (Narbonne et al., 2015, 2017; Robinson-Thiewes et al., 2021) or 48 hours for A2. For live imaging, the resulting hermaphrodites were immobilized in a 0.1% tetramisole (Sigma, L9756) M9 solution and mounted on a 3% agarose M9 pad.

Differential interference contrast (DIC) and epifluorescence images were acquired at 1 µm z-intervals using a Plan-Apochromat 20× dry objective (NA 0.8) mounted on an inverted Zeiss Axio Observer.Z1 microscope. For epifluorescence ( Fig. 1G- H), samples were excited by a LED module at 488 nm (50% intensity, 100 ms exposure) and emission was collected at 500–550 nm. Stitching and deconvolution were performed using the Zen 3.8 software. For the high-resolution confocal fluorescence acquisition shown as a pseudo-DIC overlay in Fig. 1F (the P *lim-7* :: GOA-1 ::GFP rescue image), coverslips were sealed with VALAP (1:1:1 Vaseline, lanolin, paraffin), and a single focal plane was acquired using a Leica Microsystems Stellaris 5 confocal microscope equipped with an HC PL APO CS2 63×/1.30 numerical aperture oil immersion objective. Samples were excited by a 488 nm laser (5% intensity) and emission was collected at 507–528 nm with a pinhole setting of 1. Specimens were digitally straightened using ImageJ.

**Arrested oocyte quantification**

For *fog-1 (lf),* late-L4 hermaphrodites were upshifted from 15 °C to 25 °C and maintained at this temperature along with their F1 progeny for 3 days to prevent spermatogenesis (Barton & Kimble, 1990). The resulting late-L4 feminized F1 hermaphrodites were transferred onto fresh plates at 25 °C based on vulval morphology (Seydoux et al., 1993) and allowed to develop for another 24 hours to obtain A1. For the *fog-1* (ø) *goa-1 (lf)* strain, non-fluorescent late-L4 hermaphrodites were picked from asynchronous cultures at 15 °C onto fresh plates at 25 °C. In both cases, the resulting feminized A1 were collected, immobilized in a 0.1% tetramisole (Sigma, L9756) M9 solution and mounted on a 3% agarose M9 pad. Oocyte number per gonad arm was quantified by DIC microscopy by counting morphologically distinct diakinesis-stage oocytes extending distally from the spermatheca.

**GSC mitotic index**

The progenitor zone (PZ)/GSC mitotic index (MI) was assessed following a previously established protocol (Crittenden et al., 2006; Robinson-Thiewes et al., 2021). In brief, A1 hermaphrodites prepared as above were picked into a drop of 1X PBS placed on a coverslip and rapidly dissected using a surgical needle. The coverslip was then flipped onto a poly-L-lysine-coated slide and subjected to a standard -80 °C freeze-crack procedure. Samples were subsequently fixed in -20 °C methanol for 1 minute and post-fixed in a 3.7% paraformaldehyde solution (3.7% paraformaldehyde, 1X PBS, 0.08 M HEPES, 1.6 mM MgSO4, 0.8 mM EGTA, pH 7.4) for 30 minutes. After fixation, gonads were washed twice (10 minutes each) in PBST (PBS + 0.1% Tween 20) and then blocked in PBST + 3% BSA for 1 hour at room temperature. Primary antibody staining was performed overnight at 4 °C using polyclonal rabbit anti- WAPL-1 (1:500, Sdix #4930.00.02) to label GSCs and their proliferative progeny, along with mouse monoclonal anti-phospho[Ser10]-histone H3 antibodies (1:250, Cell Signaling #9706) to mark G2/M-phase nuclei (Kocsisova et al., 2018). The samples were washed three times (10 minutes each) in PBST before incubation with A488-conjugated goat anti- mouse (Cat# A-11029; RRID:AB138404) and A546-conjugated goat anti-rabbit (Cat# A-11035; RRID:AB143051) (1:500 each) secondary antibodies for 1 hour at room temperature. Slides were washed three times with PBST and briefly stained with 0.7 µg/mL 4′,6-diamidino-2-phenylindole (DAPI) to visualize all nuclei. Vectashield™ mounting medium (Vector labs #H1900) was then applied, and the coverslip was sealed with nail polish. Prepared slides were stored at -20 °C until they were imaged. PZ nuclei counting in 3 dimensions was partially automated using an ImageJ plugin developed by Dr Jane Hubbard's laboratory (Korta et al., 2012).

**Differentiated germline tumour scoring**

Strains were maintained at 25 °C, and staged by picking GFP negative *goa-1 (lf); oma-1 (ø); oma-2 (lf)* homozygous late-L4s to a new plate at 25 °C. The resulting animals were imaged 48 hours later (at A2). We classified individuals as tumorous if they contained obviously more oocyte-like cells than same-age stable hyperplastic *oma-1* (ø); *oma-2 (lf)* controls (Clouet et al., 2025). Disorganized oocyte-like cells usually fill the uterus of tumorous animals but not of controls, facilitating the scoring of this phenotype.

**Statistical analysis**

All bar/dot plots were generated using GraphPad Prism 10.0. For each dataset, normality was verified using the Shapiro-Wilk test, and variance equality was assessed using the Brown-Forsythe test for multi-group comparisons. Tests were chosen according to the following criteria. For multi-group comparisons where all distributions were normal and variances equal, the one-way ANOVA with Tukey's multiple comparisons was used. When distributions were non-Gaussian, the Kruskal-Wallis test with Dunn's multiple comparisons was applied instead. Tests used for each dataset are indicated in the figure legend.

## Reagents

**Table:** 

**Strain/** **plasmid name**	**Name in text/ description**	**Genotype/ source material**	**Source/ oligo sequences** **(5'-> 3')**
N2	wild-type (WT)	wild-type	*Caenorhabditis* Genetics Center (Brenner, 1974)
JK3743	*fog-1 (ø) *	GFP-negative progeny of *fog-1 ( q785 )/ hT2 [ bli-4 ( e937 ) let-?( q782 ) qIs48 ] (I;III) *	*Caenorhabditis* Genetics Center (Morgan et al., 2010)
JK560	*fog-1 (lf) *	*fog-1 ( q253 )ts I *	*Caenorhabditis* Genetics Center *(Barton & Kimble, 1990)*
MT2426	*goa-1 (lf) *	*goa-1 ( n1134 ) I *	*Caenorhabditis* Genetics Center
KG524	*gsa-1 (gf) *	*gsa-1 ( ce94 )gf I *	(Castaneda et al., 2020)
UM307	*fog-1 (ø) goa-1 (lf) *	GFP-negative progeny of *fog-1 ( q785 ) goa-1 ( n1134 )/ hT2 [ bli-4 ( e937 ) let-?( q782 ) qIs48 ] (I;III) *	(Narbonne et al., 2015)
UTR498	*gsa-1 (gf) fog-1 (lf) *	*gsa-1 ( ce94 )gf fog-1 ( q253 )ts I *	This study
UTR873	* sheath:: GOA-1 (+) *	*narEx166 [pCAM37(P lim-7 :: GOA-1 N-term::GFP:: GOA-1 C-Term); Pmyo-3::mCherry] *	This study; see primer info at the end of table (Frøkjær-Jensen et al., 2008; Kumar et al., 2021)
UTR874	*fog-1 (ø) goa-1 (lf); sheath:: GOA-1 (+) *	*fog-1 ( q785 ) goa-1 ( n1134 )/ hT2 [ bli-4 ( e937 ) let-?( q782 ) qIs48 ] (I;III); narEx166 [pCAM37(P lim-7 :: GOA-1 [N-term]::GFP:: GOA-1 [C-Term]); Pmyo-3::mCherry] *	This study, (Frøkjær-Jensen et al., 2008; Kumar et al., 2021)
LX2060	* P goa-1 :: GOA-1 ::GFP *	*vsSi32 [P goa-1 :: GOA-1 ::GFP + unc-119 (+)] III. *	(Kumar et al., 2021)
UTR508	*fog-1 (lf), P goa-1 :: GOA-1 ::GFP *	*fog-1 ( q253 )ts I, vsSi32 [P goa-1 :: GOA-1 ::GFP + unc-119 (+)] III. *	This study
UTR19	*oma-1 (ø); oma-2 (lf) *	*cpSi42 [Pmex-5::mNeonGreen::PLCδ-PH:: tbb-2 3'UTR + unc-119 (+)] II; oma-1 ( zu405te33 )/ nT1 [ qIs51 ] IV; oma-2 ( te51 )/ nT1 V *	(Clouet et al., 2025)
UTR426	*goa-1 (lf); oma-1 (ø); oma-2 (lf) *	*goa-1 ( n1134 ) I; cpSi42 [Pmex-5::mNeonGreen::PLCδ-PH:: tbb-2 3'UTR + unc-119 (+)] II; oma-1 ( zu405te33 )/ nT1 [ qIs51 ] IV; oma-2 ( te51 )/ nT1 V *	This study
pCAM37	* P lim-7 :: GOA-1 N-Term::GFP:: GOA-1 C-Term *	LX2060 gDNA for the insert; pXA9 (derived from pGC240 (Voutev et al. 2008) for the backbone.	Insert forward: TTGGAGGGTACCGGTAGAAAAAATGGGTTGTACCATGTCACAGGA Insert reverse: AGGGGAAACAAAATGAAGAGAATTTAATACAAGCCGCATCCACGAAG Backbone forward: CTTCGTGGATGCGGCTTGTATTAAATTCTCTTCATTTTGTTTCCCCT Backbone reverse: TCCTGTGACATGGTACAACCCATTTTTTCTACCGGTACCCTCCAA
*E. coli*	*OP50*	* Standard E. coli bacteria used as C. elegans food *	*Caenorhabditis* Genetics Center

**Table 1.** *C. elegans* strains and reagents used in this study.
